# Use of a Novel Training Aid for Teaching the Nursing Care of Central Venous Catheters

**DOI:** 10.31486/toj.25.0042

**Published:** 2025

**Authors:** Michael D. Smith, Christie Poindexter, Ashlee Ellington, Richard Guthrie

**Affiliations:** ^1^ Ochsner Clinical Simulation and Patient Safety Center, Ochsner Clinic Foundation, New Orleans, LA; ^2^ The University of Queensland Medical School, Ochsner Clinical School, New Orleans, LA; ^3^ Center for Quality and Patient Safety, Ochsner Clinic Foundation, New Orleans, LA

**Keywords:** *Bandages*, *central venous catheters*, *nursing*, *sepsis*, *simulation training*

## Abstract

**Background:**

The occurrence of central venous catheter infections is a metric that hospital systems track. We determined that central line–associated bloodstream infections (CLABSIs) at our institution occurred in a delayed fashion, prompting us to raise the question of whether the infections were related to insertion or to catheter care and then to design a training simulation focused on how to change the dressing for central venous catheters.

**Methods:**

Using low-cost equipment, such as refrigerator magnets and tape, we constructed a reusable SorbaView SHIELD Contour (Centurion Medical Products Corporation) sterile central line dressing for use in training.

**Results:**

This cost-effective simulation innovation gives staff who care for central venous catheters the opportunity to practice the manual skills involved in dressing changes and eliminates the problem of expending a single-use dressing with each learner experience. The magnetic dressings can be reused as long as the integrity of the SorbaView SHIELD Contour is preserved.

**Conclusion:**

We hope that ongoing training with this simulation model, along with demonstration of competency, will result in standardized central line care and a decrease in CLABSI rates at our institution.

## INTRODUCTION

Central venous catheters (CVCs) are vascular access devices commonly used in the care of critically ill patients, especially those requiring vasopressor support. However, a challenge associated with central venous catheter use is the potential for development of central line–associated bloodstream infections (CLABSIs) that are linked to increased morbidity and mortality risks^1^ and additional costs/length of stay.^2^ We determined that the majority of CLABSIs at our institution were delayed and thus likely attributable to the care of the catheter rather than the actual insertion of the catheter.^3^ While much training has been devoted to the sterile insertion of the catheter, we sought to improve nursing training in the care of the catheter site.

We know that hands-on simulation instruction followed by debriefing is an effective teaching approach that results in more knowledge retention compared to a traditional didactic lecture.^2^ However, the $153.91 (USD) price of 50 SorbaView SHIELD Contour (Centurion Medical Products Corporation) sterile central line dressings was cost-prohibitive for training use, thus limiting the nurses’ training to verbal instruction only. To address the lack of hands-on instruction, we developed a magnet-based CVC dressing change trainer that can be reused. With our dressing change trainer, nurses can practice proper technique without the institution incurring the high costs associated with single-use dressings during training.

## METHODS

### Central Venous Catheter Dressing Change Trainer Materials Required

The Table lists the materials required to construct the CVC dressing change trainer and provides approximate costs and sources for each component.

**Table.** Materials, Approximate Costs, and Sources for the Central Venous Catheter Dressing Change Trainer

Item	Quantity	Approximate Cost, $	Source
Gen II Ultrasound Central Line Training Model	1	3,451.01^a^	Elevate Healthcare (item# BPH663)
6-mm × 3-mm circular refrigerator magnets	19	11.99/200 magnets, 0.06/each	Amazon
4" × 4-3/4" Tegaderm transparent film dressing	2	1.21, 0.605/each	3M Company
2-3/8" × 2-3/4" Tegaderm transparent film dressing	3	0.61, 0.204/each	3M Company
SorbaView SHIELD Contour dressing	1	3.08	Centurion Medical Products Corporation
Office tape	1 roll	3.00	3M Company
8-mm biopsy punch	1	1.65	Integra Miltex
Moulage gel in color that matches manikin	1	13.75	MEDICFX
Liquid makeup foundation in color to match manikin	1	11.99	Maybelline New York
Pen	1	1.00	Paper Mate
Cyanoacrylate (super glue)	1	3.64	Loctite
Total		51.92	

^a^Because our institution already owned the central line training model and did not have to purchase it for this simulation, the cost of the manikin is not included in the total.

### Central Venous Catheter Dressing Change Trainer Construction Steps

**Figure 1.**
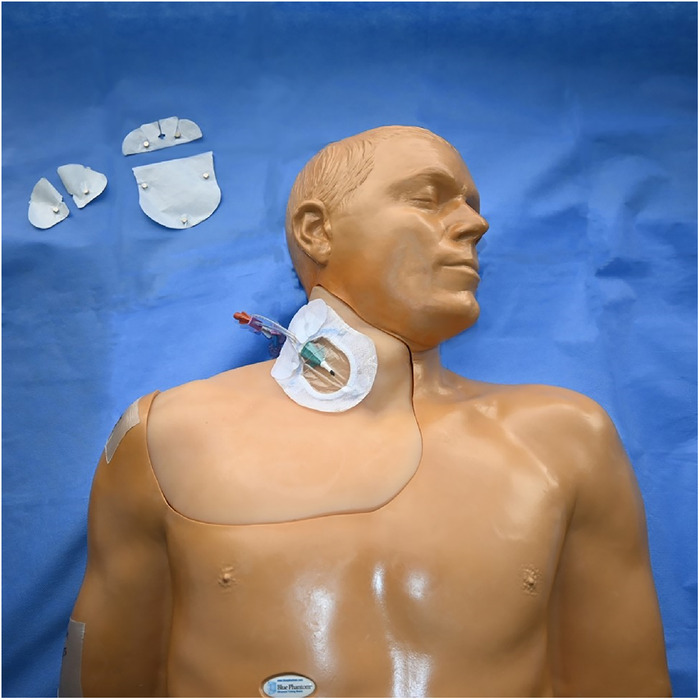

**Figure 2.**
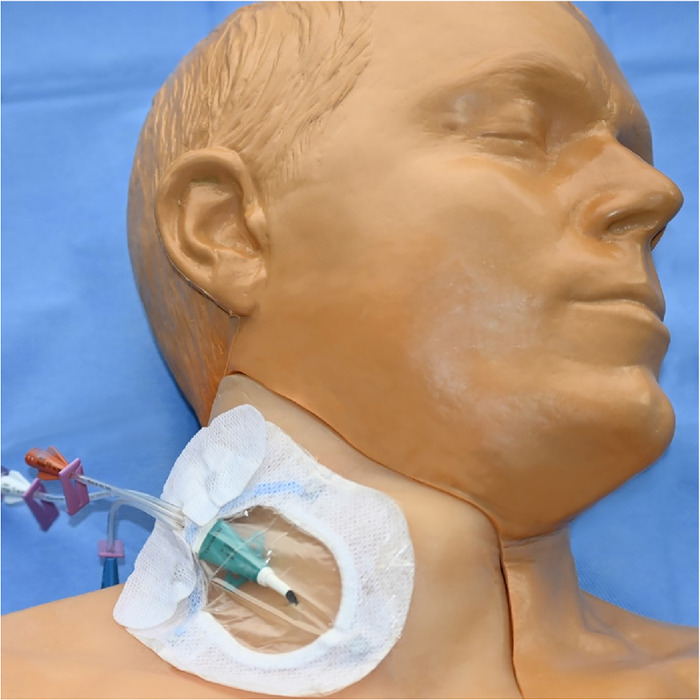

Figures 1 and 2 show the completed central venous catheter dressing change trainer in place on the central line training manikin in the simulation center. Illustrated step-by-step instructions for constructing the trainer are provided in this section.

**Figure 3.**
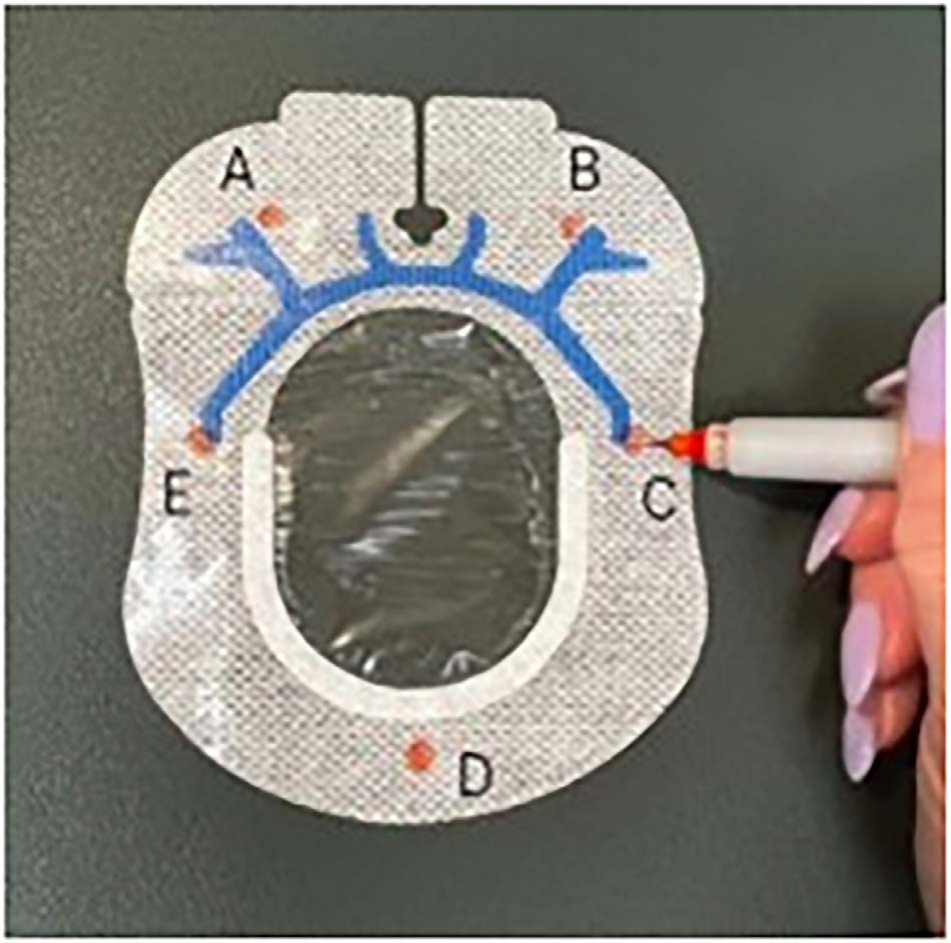
  - Place one 6-mm × 3-mm circular refrigerator magnet on each of the 5 marked sites (Figure 4). When placing the magnets on the dressing, ensure magnets are oriented with matching polarity.

1. *Preparing the Adhesive Sides of the Dressing Pieces*
  - Peel off the 2 backing pieces of the larger of the 2 SorbaView SHIELD Contour dressing pieces to expose the adhesive side. Do not discard the backing pieces.
  - Use a pen to mark 5 magnet placement sites (A through E) on the circumference of the adhesive side of the SorbaView SHIELD Contour dressing (Figure 3).

**Figure 4.**
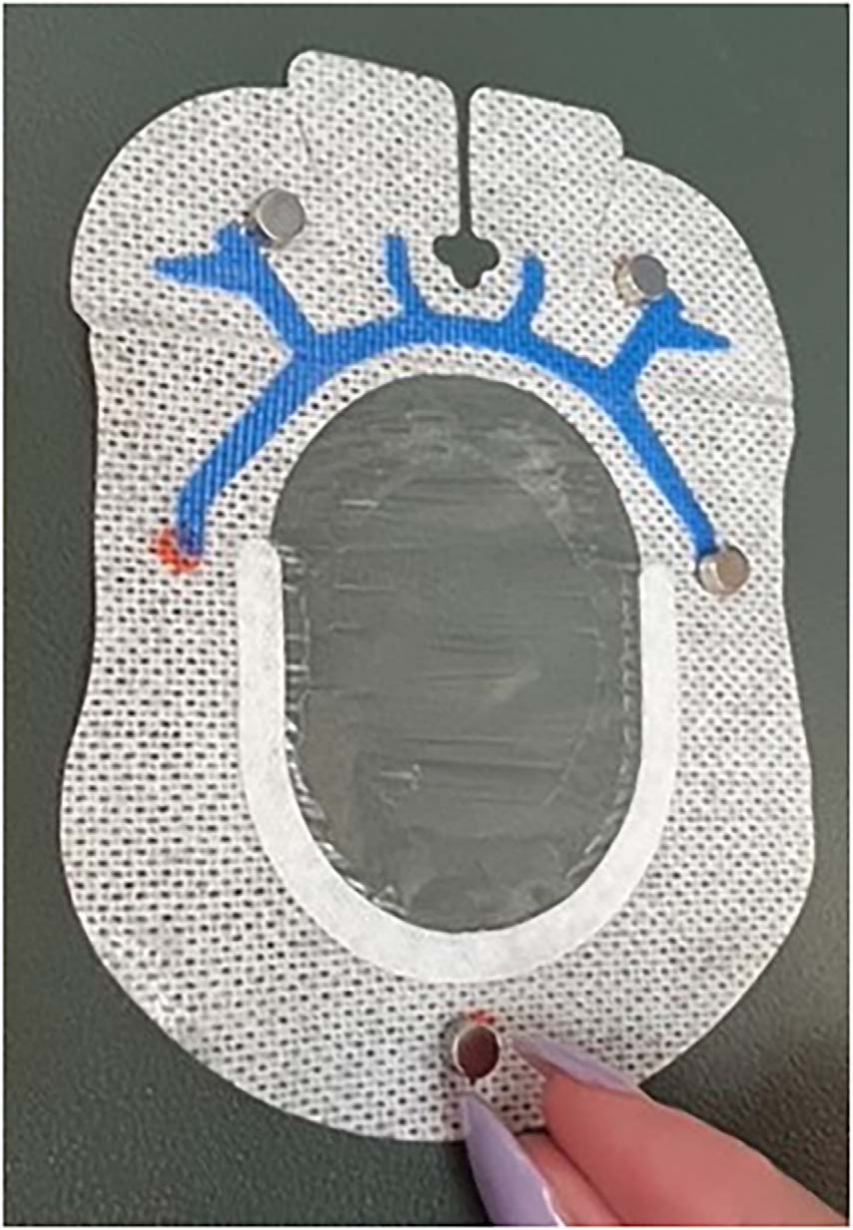
  - Use small but appropriately sized pieces of office tape to secure the magnets to the dressing (Figure 5).

**Figure 5.**
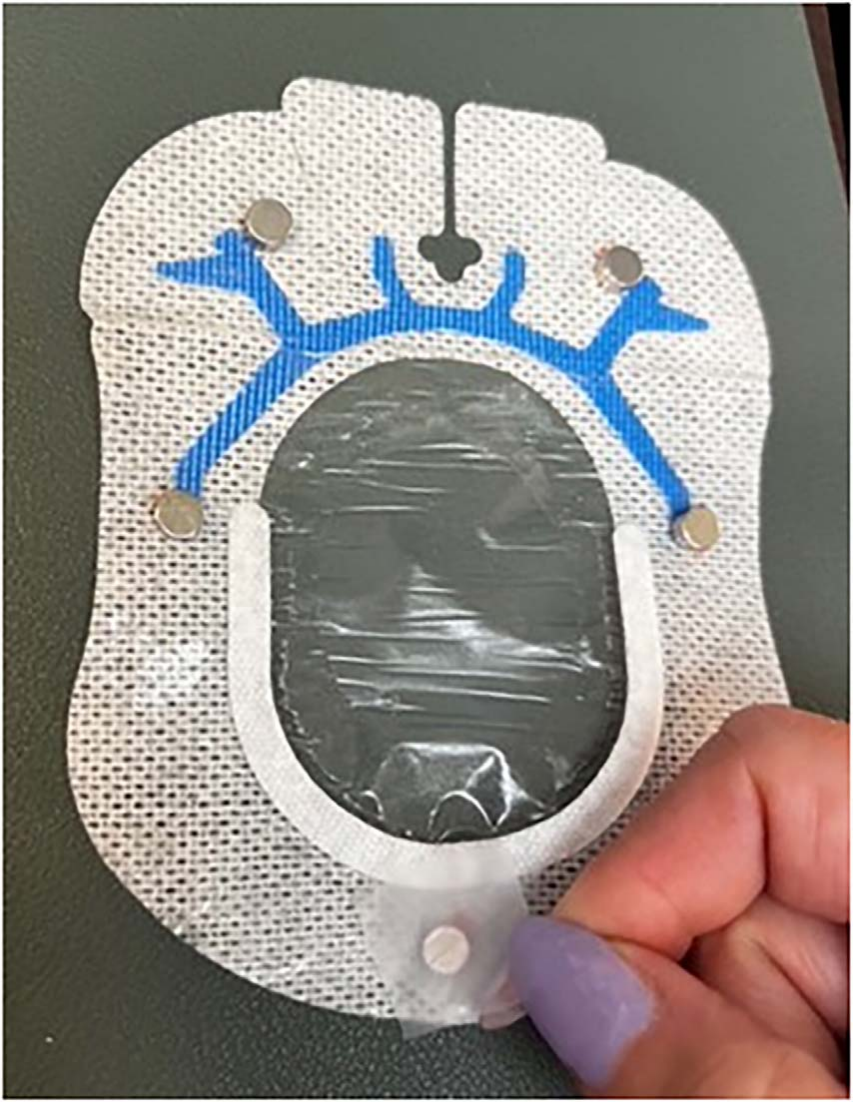
  - Cover the dressing and magnets with a 4" × 4-3/4" Tegaderm transparent film dressing (Figure 6). Pull off the paper tab and trim any excess film.

**Figure 6.**
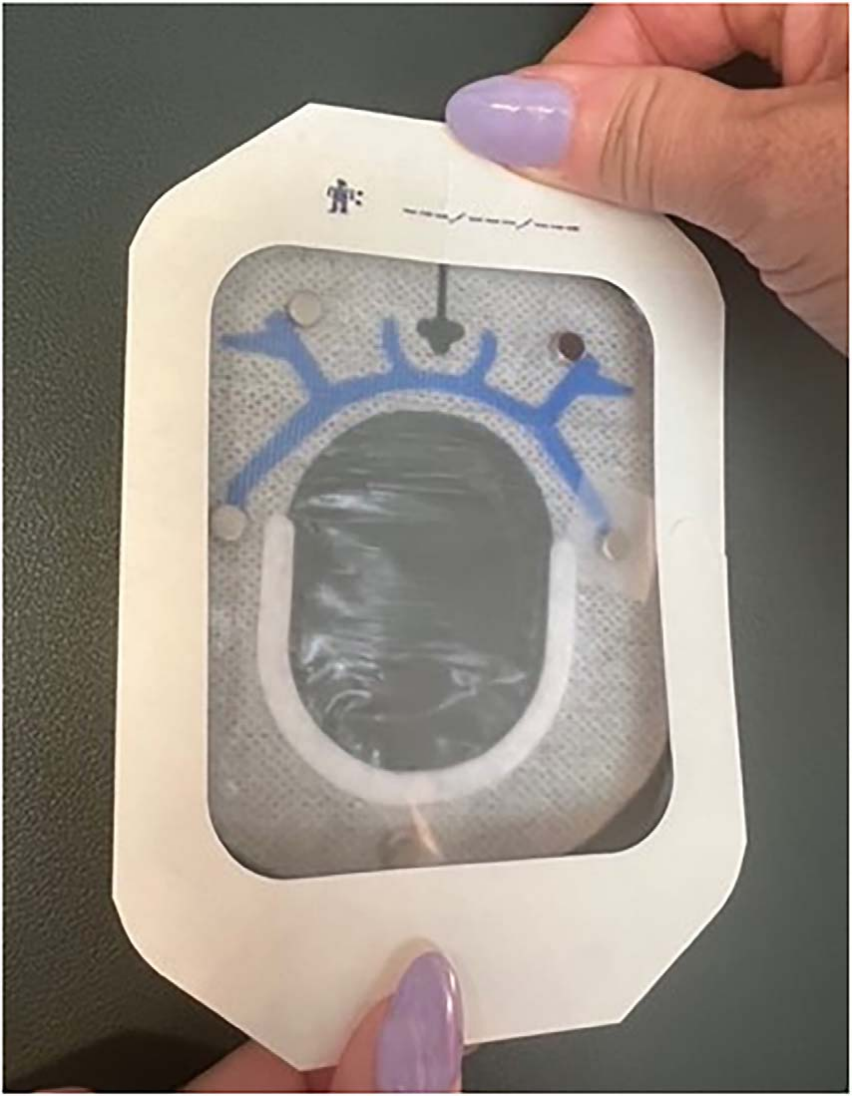
  - Peel off the 2 backing pieces of the smaller of the 2 SorbaView SHIELD Contour dressing pieces to expose the adhesive side. Do not discard the backing pieces.
  - With the formerly adhesive side of the larger dressing piece facing up, align the holes of both dressings, ensuring the adhesive side of the smaller dressing piece is also facing up (Figure 7). The smaller dressing piece should be over magnet placements A and B of the larger dressing piece.

**Figure 7.**
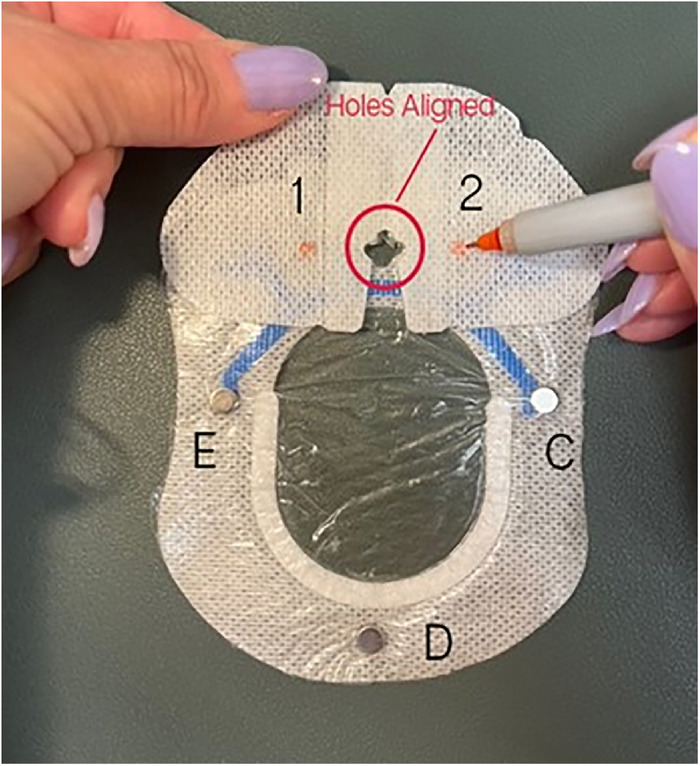
  - Use a pen to mark 2 magnet placement sites (1 and 2) on the adhesive side of the smaller dressing piece so that site 1 is directly on top of site A and site 2 is directly on top of site B (Figure 7).
  - Place one 6-mm × 3-mm circular refrigerator magnet on each magnet placement site on the smaller dressing and secure with office tape (Figure 8). Ensure magnets are oriented with the same polarity as the larger dressing.

**Figure 8.**
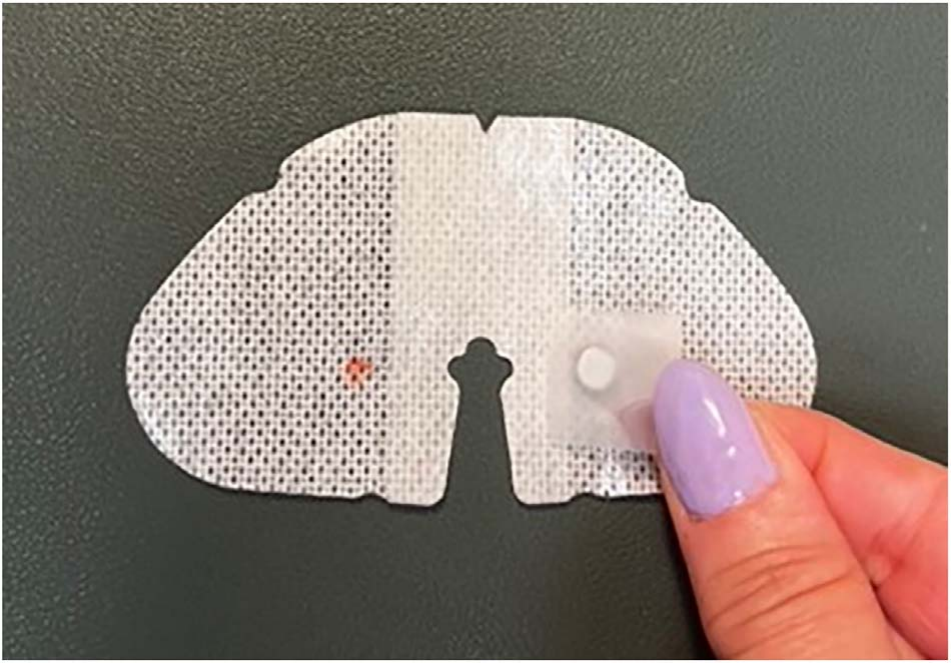
  - Cover the dressing and magnets with a 2-3/8" × 2-3/4" Tegaderm transparent film dressing (Figure 9). Pull off the paper tab and trim any excess film.

**Figure 9.**
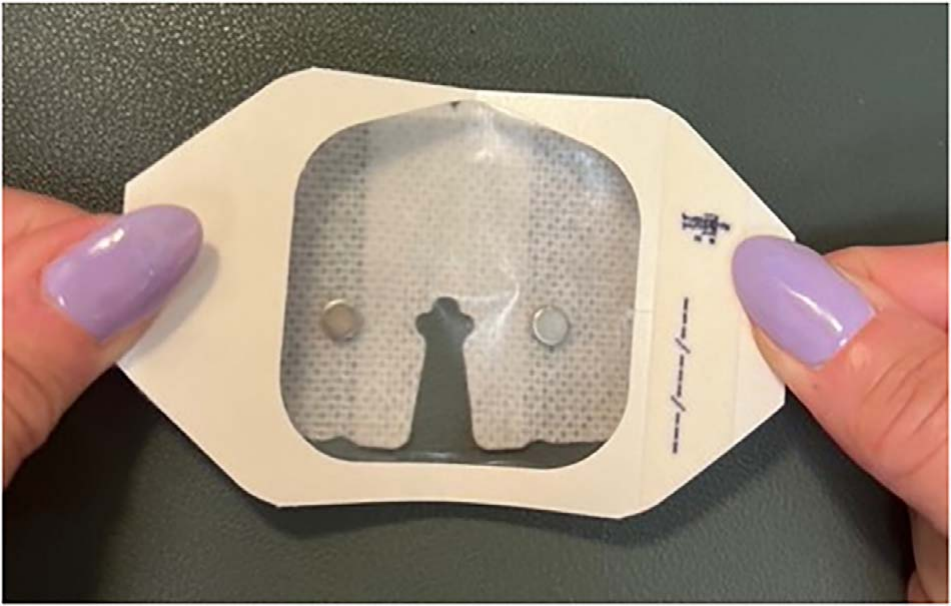
2. *Preparing the Backing Sides of the Dressing Pieces*
  - Use an emery board nail file to buff off the waxy topcoat of the unbranded side of all 4 backing pieces (Figure 10). This step results in better adhesion of the magnets.

**Figure 10.**
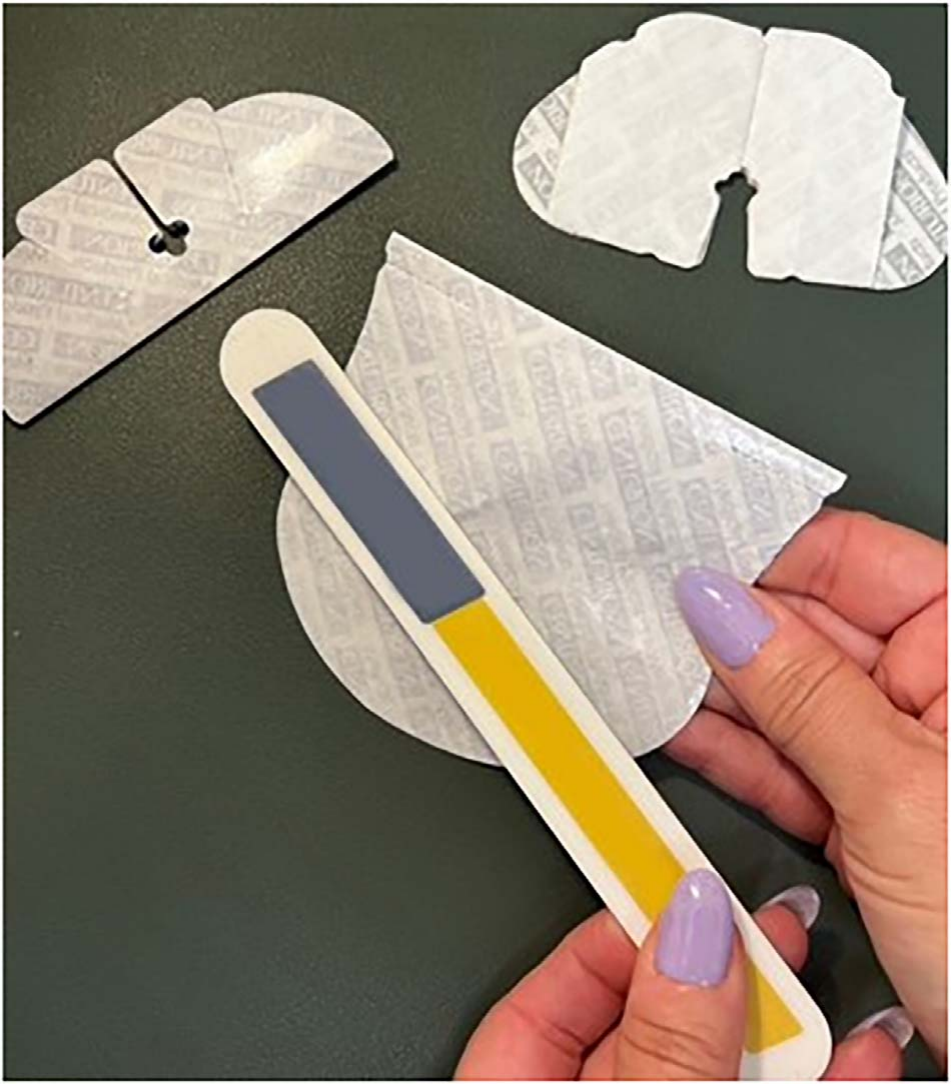
  - With the nonadhesive side of the larger of the 2 SorbaView SHIELD Contour dressing pieces facing up, align the 2 backing pieces on top in their original orientation, ensuring the freshly buffed sides are facing up (Figure 11). The smaller backing piece should be over magnet placements A and B of the dressing.

**Figure 11.**
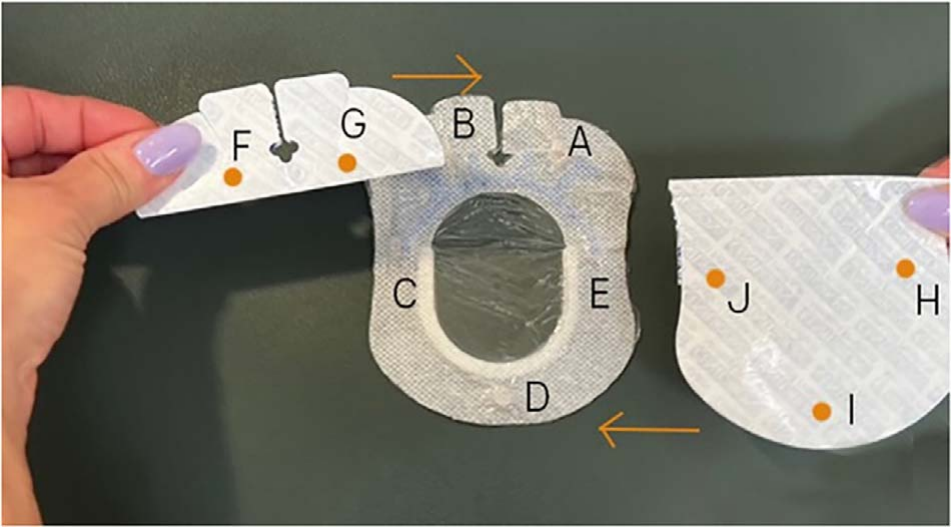
  - Use a pen to mark 5 magnet placement sites (F through J) on the buffed side of the 2 backing pieces so that site F is directly on top of site B, site G is on top of site A, site H is on top of site E, site I is on top of site D, and site J is on top of site C (Figure 11).
  - Place one 6-mm × 3-mm circular refrigerator magnet on each marking, ensuring magnets are oriented with the same matching polarity as all previous magnets, and secure with office tape.
  - Place a 4" × 4-3/4" Tegaderm transparent film dressing over the magnets and buffed backing of the larger of the 2 backing pieces to secure magnets. Pull off the paper tab and trim any excess film.
  - Place a 2-3/8" × 2-3/4" Tegaderm transparent film dressing over the magnets and buffed backing of the smaller of the 2 backing pieces to secure magnets. Pull off the paper tab and trim any excess film.
  - With the nonadhesive side of the smaller of the 2 SorbaView SHIELD Contour dressing pieces facing up, align the 2 backing pieces on top in their original orientation, ensuring the freshly buffed sides are facing up (Figure 12). The backing pieces should be over magnet placements 1 and 2 of the smaller dressing.

**Figure 12.**
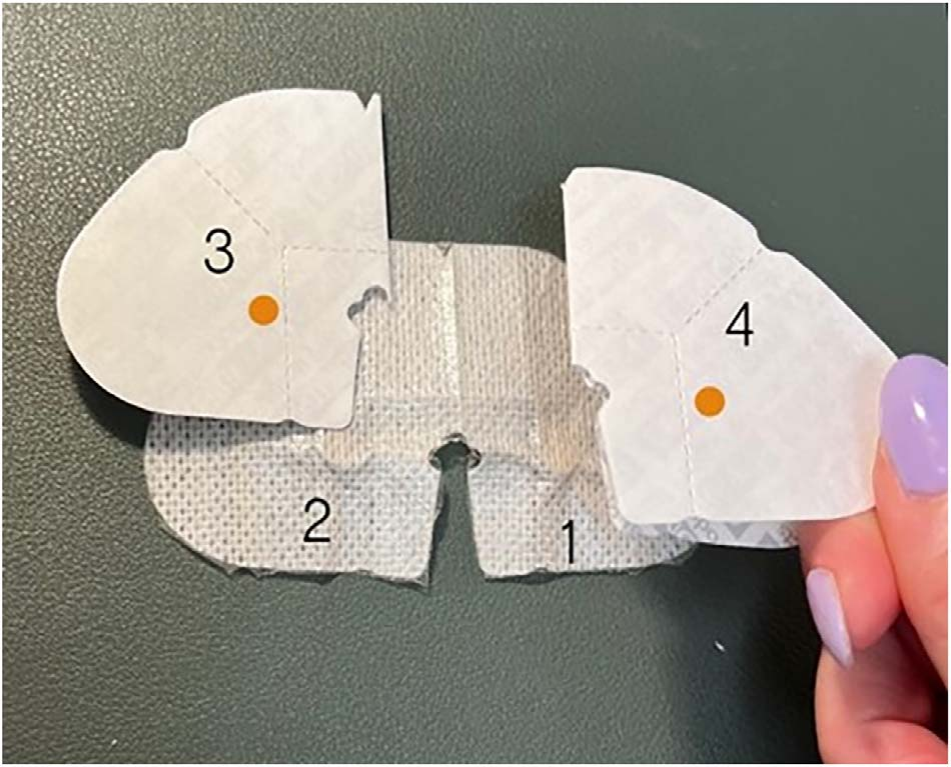
  - Use a pen to mark 2 magnet placement sites (3 and 4) on the buffed side of the dressing so that site 3 is directly on top of site 2 and site 4 is on top of site 1.
  - Place one 6-mm × 3-mm circular refrigerator magnet on each marking, ensuring magnets are oriented with the same matching polarity as all previous magnets, and secure with office tape.
  - Place a 2-3/8" × 2-3/4" Tegaderm transparent film dressing over the magnets and backing for the smaller dressing. Pull off the paper tab and trim any excess film.
  - All components of the dressings should now attach to each other.
3. *Preparing the Task Trainer*
  - Place the larger of the 2 dressing pieces in the appropriate place on the central line training manikin (Figure 13).

**Figure 13.**
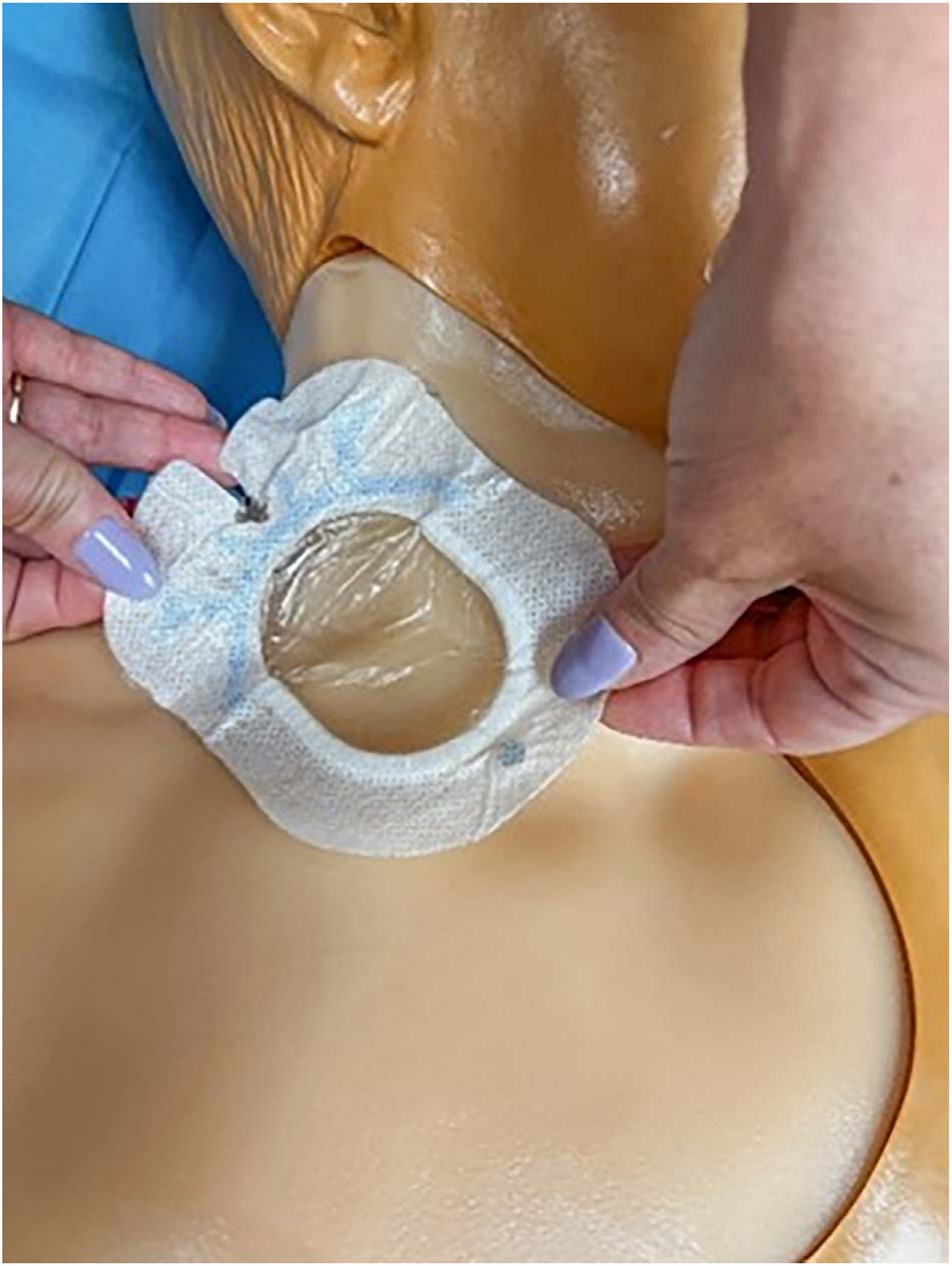
  - Use a pen to mark 5 magnet placement sites on the manikin (Figure 14).

**Figure 14.**
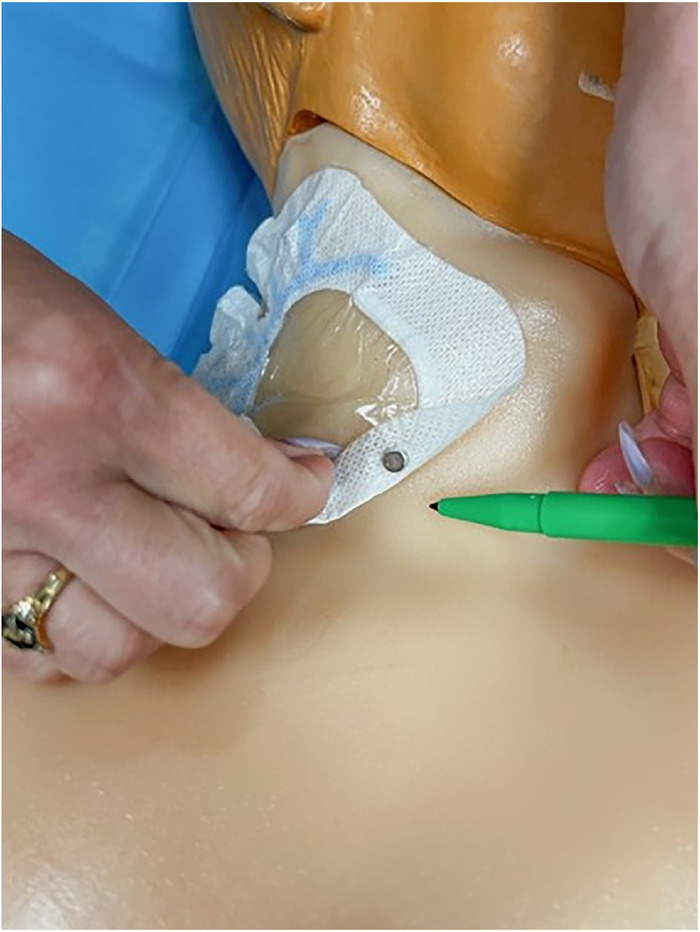
  - Use an 8-mm biopsy punch to make holes where the marks on the manikin are.
  - Put a drop of cyanoacrylate in each hole and place one 6-mm × 3-mm circular refrigerator magnet in the hole, ensuring magnets are oriented with the same matching polarity as all previous magnets.
  - Cover the holes and magnets with pieces of office tape to help the magnets set appropriately in cyanoacrylate.
  - Use moulage gel and a dab of liquid makeup foundation to cover the magnets and blend with the color of the manikin.

## RESULTS

This innovation in simulated central line dressing care eliminates the problem of expending a single-use dressing with each learner experience. The magnetic dressing can be reused as long as the integrity of the SorbaView SHIELD Contour dressing is preserved. To help with the preservation of this reusable dressing, we recommend placing an additional Tegaderm transparent film dressing on the top sides of both SorbaView SHIELD Contour pieces.

One change planned for future iterations of this model is to use larger plate magnets in the central line training manikin to make application of the dressing easier (rather than having to match small magnet to small magnet).

## DISCUSSION

This innovation allows the learner to complete the tactile tasks involved with central line care at a lower cost per education session and has the potential to help reduce the problem of delayed CLABSI. We plan to study the impact of this innovation on CLABSI occurrence at our institution.

## CONCLUSION

We hope to reduce CLABSI rates by increasing hands-on training through required onboarding simulation courses for individuals who care for central lines. The goal of this approach is to lower the morbidity, mortality, hospital stays, and health care costs associated with CLABSIs.

## Notes

*This article meets the Accreditation Council for Graduate Medical Education and the American Board of Medical Specialties Maintenance of Certification competencies for Patient Care, Medical Knowledge, and Practice-Based Learning and Improvement.*
